# A brief history of masting research

**DOI:** 10.1098/rstb.2020.0423

**Published:** 2021-12-06

**Authors:** Walter D. Koenig

**Affiliations:** ^1^ Hastings Natural History Reservation, University of California Berkeley, Carmel Valley, CA 93924, USA; ^2^ Cornell Lab of Ornithology, Cornell University, Ithaca, NY 14850, USA

**Keywords:** masting behaviour, mast fruiting, mast seeding, pannage, spatial synchrony, variable seed production

## Abstract

Although it has long been recognized that seed production by many forest trees varies greatly from year to year, masting (along with ‘mast fruiting’, ‘mast seeding’ and ‘masting behaviour’) as a concept referring to such variability is a relatively recent development. Here, I provide a brief history of masting research, highlighting some of the early contributions by foresters, zoologists and others that paved the way for the burgeoning number of studies currently being conducted by researchers around the world. Of particular current interest is work attempting to understand the proximate mechanisms, evolutionary drivers and community effects of this important ecological phenomenon as well as the ways that climate change may influence masting behaviour in the future.

This article is part of the theme issue ‘The ecology and evolution of synchronized seed production in plants'.

## 1. Introduction and early history (pre-1970)

**Figure 1.**
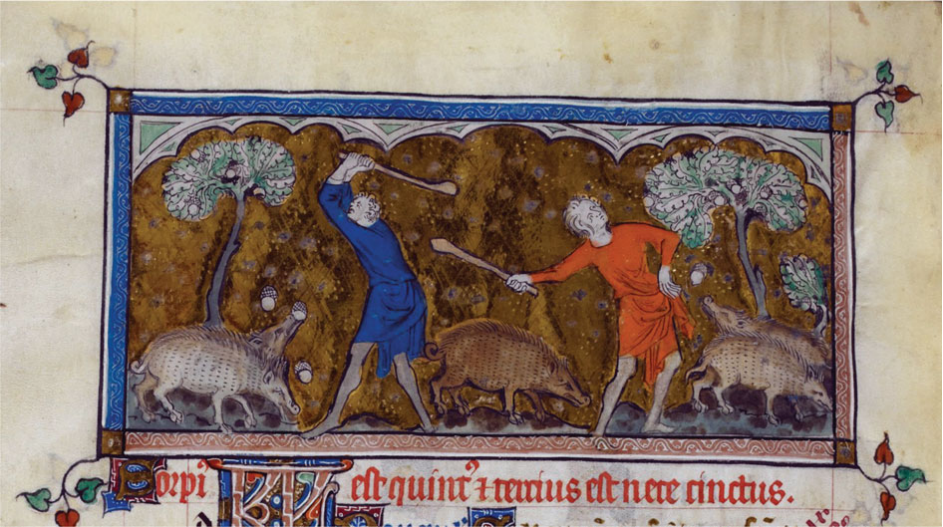
Men knocking down acorns to feed swine. From the *Queen Mary Psalter*, made in England between 1310 and 1320. Image in the public domain courtesy of the British Library.

**Figure 2.**
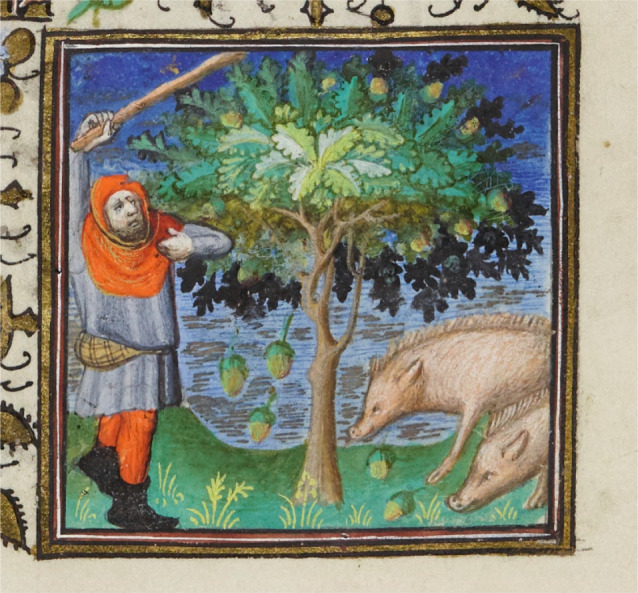
Another illustration of a man knocking down acorns to feed swine. From a *Book of Hours*, made in France in the early 15th century. Beating of oaks to knock down acorns was commonly used to illustrate the ‘Labour of the Month’ for November, when pigs were fattened prior to slaughter in December. Image in the public domain courtesy of Trinity College, Cambridge.

Historically, the seeds of forest trees have been an important human food source in many parts of the world, including Southeast Asia, North Africa, and both North and South America. In North America, for example, acorns—seeds of oaks (*Quercus* spp.)—made up a substantial portion of the diet of some tribes of Native Americans, who succeeded in overcoming the considerable time and effort required to collect, process and store them [1–3]. Acorns have historically also been a relatively common food source in many parts of Europe [4]. Nonetheless, reference to the seeds of forest trees in medieval Europe has more commonly been associated with *pannage*—the practice of fattening domestic pigs under oak (*Quercus* spp.) and beech (*Fagus* spp.) in exchange for a fee [5–7]. Pannage being a common peasant activity in the autumn (figures 1 and 2), mast has thus not always been viewed favourably. For example, in *De rerum natura* (On the Nature of Things), a poem written by the Roman philosopher Lucretius in the first century B.C., Lucretius touts the technological advances of shelter and fire, claiming that they facilitated monogamy and the emergence of social communities and displaced promiscuous earlier societies in which indiscriminate fornication and the trading of sex for acorns were rampant [8]. (To the best of my knowledge, this is no longer a common practice.) Feeding pigs with the mast of acorns is still widespread in several countries, especially the *dehesas* of Spain and Portugal, but also, at least until recently, parts of France, England and the United States [9,10].

Referring to acorns and other seeds of forest trees as ‘mast’ was well established by the Middle Ages. The Oxford English Dictionary lists early references going back at least as far as the illuminated Byzantine Codex *Paris Psalter,* produced in Constantinople in the mid-tenth century. More recent references include mentions by Chaucer around 1380 [11], Shakespeare in the 1623 play *Timon of Athens* [12] (The Oakes beare Mast, the Briars Scarlet Heps), and Jonathan Swift's 1726 classic, *Gulliver's Travels* [13]. (Gulliver, touring the grand academy of Lagado, describes the locals' way of ploughing whereby they bury a ‘quantity of acorns, dates, chestnuts, and other mast or vegetables’ in the field, after which a large a number of hogs are driven into the field. The hogs then ‘root up the whole ground in search of their feed, and make it fit for sowing, at the same time manuring it with their dung’).

By contrast, the history of ‘masting’ (along with the more descriptive ‘masting behaviour’, ‘mast fruiting’ and ‘mast seeding’) as referring to the variable and synchronized production of seeds by a population of plants [14], rather than putting swine out to feed on acorns, is relatively recent. This is despite it having been long obvious to both foresters and farmers that seed crops of beech, oak and various other species varied considerably from year to year. One of the first of the former to consider the causes of masting was Georg Ludwig Hartig (1764–1837), a renowned German forester whose career included being Chief Inspector of Forests in Stuttgart and Berlin and culminated with an Honourary Professorship at the University of Berlin in 1830. Hartig suggested that periodic fruiting by European beech (*Fagus sylvatica*) was related to the period of time required by trees to build up reserves depleted during a large mast year [15], foreshadowing the resource threshold model that has shaped thinking about masting over the past 20 years.

Scientific advances concerning the ecology of masting behaviour during the first half of the twentieth century were made by both botanists and zoologists. Noteworthy among the latter was Charles Elton (1900–1991), whose broad ecological interests included population cycles. Recognizing the role of seed production by forest trees as a potential factor in such cycles, Elton discussed the effects of climate as a driver of mast years and, in a 1924 discussion of mouse plagues and other decadal-length population cycles, suggested

The scattered notes on the subject [of mast crops] in the phenological reports of the *Quarterly Journal of the Meteorological Society* show that there has been an unusually heavy beech-mast crop in Britain every eleven years … . This periodicity in beech crops is suggestive, but there are not sufficient records to prove the hypothesis that the crops are correlated with the sunspot cycle [16, p. 143].

This is apparently the first and, until recently, last time that sunspots have been proposed as a driver of masting behaviour. This hypothesis has been freshly revived in a paper proposing that reproductive output by tropical trees is driven by solar-wind energy flux in the Earth's magnetosphere [17], a phenomenon related to sunspot activity [18].

Of the botanists considering the phenomenon of masting, two are of special note. First is Georg Albrecht Klebs (1857–1918), who suggested in 1903 that mast seeding was associated with years when more resources were available—what is now referred to as the resource matching hypothesis [19]. Second is Edward Salisbury (1886–1978), who called attention to the biological importance of periodic mast fruiting as a means of overwhelming seed predators in mast years—the predator satiation hypothesis—as early as 1942 [20]. Foresters discussing mast fruiting by trees in this era included Jacob Roeser in the United States, whose 1942 paper discussed the influence of climate on seed production of Douglas fir (*Pseudotsuga menziesii*) [21], and Antii Reinikainen in Finland. Drawing upon speculation published in 1910 by Joseph Whitaker in Britain [22], Reinikainen's 1937 paper summarized cone crop data throughout Finland, supporting the hypothesis that cone crop failures over large geographic regions drive irruptive migrations of red crossbills (*Loxia curvirostra*) [23].

Thus, by the second half of the twentieth century, both major components of mast fruiting—variability and synchrony—were implicitly, if not explicitly, recognized, and resources, weather and predation (along with sunspots) had all been suggested as playing roles in its ecology. Moreover, masting was recognized as being a potential driver of several widespread ecological phenomena, including population cycles of some mammals and irruptive migrations of boreal birds. Studies published over the next 20 years furthered these ideas in various ways. In 1956, Fowells & Schubert [24] discussed evidence that resources such as fertilizers and light stimulated trees to produce large seed crops as well as the potential for freezing temperatures to kill developing conelets, thus affecting masting patterns via an ecological ‘veto’. Lauckhart [25], in a 1957 paper on the role of food in driving population cycles, proposed the existence of a latitudinal gradient in masting and animal cycles whereby both become more pronounced as one proceeds northward. (Subsequent analyses have failed to support this pattern, at least for masting: Koenig & Knops [26] found that variability in seed production decreased with latitude, while both Kelly & Sork [27] and Pearse *et al*. [28] found a nonlinear pattern with the largest variability at mid-latitudes.) Following up on Reinikainen's work, Svärdson provided an extensive discussion of seed crop variability and how it potentially drives avian irruptive migrations in a notable 1957 paper [29]. Svärdson went on to summarize data suggesting considerable synchrony in cone production by a series of boreal and more temperate tree genera, including spruce (*Picea* spp.), birch (*Betula* spp.), oak and beech, which he attributed to above-average temperatures during the summer as cones and seeds were developing. Also worthy of note during this era were numerous quantitative studies providing data key to subsequent attempts to better understand masting behaviour (i.e. [30–34]).

## 2. The modern era (1970–1980)

Although arbitrary, what can reasonably be considered the modern era of the study of masting began with the 1971 publication of Daniel Janzen's review of seed predation by animals [35]. Janzen explicitly discussed both the variability and synchrony characteristic of masting behaviour, as well as some of the potential proximate (i.e. weather) and ultimate (i.e. predator satiation) drivers of this phenomenon. He proposed that masting should be the result of selection for magnifying disruptive meteorological events by physiological systems that either render the plant hypersensitive or hyposensitive to such activity, a hypothesis highlighted recently by Fernández-Martínez *et al*. [36] and Kelly *et al*. [37]. He also raised the particularly vexing problem of the coexistence of species that require different numbers of years to mature fruits from flowers (especially pronounced in oaks), an issue that has been discussed by few subsequent authors and remains unresolved today [38–40]. Janzen went on to discuss masting in classic papers published over the next several years, including one specifically on mast fruiting in the Dipterocarpaceae [41] and another on the reproductive biology of bamboos (family Poaceae) in which mast seeding is defined for the first time:

*Mast seeding* is the synchronized production of seed at long intervals by a population of plants. The term derives from oak mast, beech mast, etc. as traditionally used to describe the large amount of acorns, beech seeds, etc. on the ground beneath midlatitude forests in a mast year [14, p. 354].

Upon close scrutiny, this definition begs several questions that have yet to be fully resolved, despite more than one attempt. (How synchronized? How long and regular an interval between mast events? How large a population? And how should a ‘large amount’ of seed be defined? [42,43]). Nonetheless, the definition remains essentially intact today. Particularly visionary was Janzen's 1978 book chapter discussing seeding patterns of tropical trees [44], which emphasized predator satiation as the driving force for mast fruiting but mentions the alternative hypothesis of pollen coupling (although not called as such), and even discusses the importance of economies of scale, a concept basic to current thinking about masting that was later brought to prominance by Norton and Kelly's 1988 paper [45].

Four additional papers are among those forming a bridge to the next era of masting investigation. First is Smith's 1970 monograph on the coevolution of pine squirrels (*Tamiasciurus*) and conifers [46], in which he points out that cone production is not a truly cyclic phenomenon, is often synchronous over large geographic regions and suggests what is now referred to as the environmental prediction hypothesis:

An equally important ultimate function…may be the production of seeds mainly during the years in which they have the best chance of competing with other vegetation when they germinate [46, p. 361].

This hypothesis has subsequently been supported primarily in the case of post-fire seed production [43], but several recent papers argue for its importance predicated on the role of rainfall in the New Zealand tree *Dysoxylum spectabile* [47], an interaction between cooling and drought in five *Shorea* species (Dipterocarpaceae) [48] and, more generally, water stress in mesic temperate forests [49].

Second is Bock and Lepthien's 1976 analysis of seed production by boreal and montane trees and boreal bird eruptions [50]. Gathering together data on cone production of boreal trees, they found support for and expanded Svärdson's earlier [29] finding of synchronous seed production, concluding boldly:

Results of this study point toward the existence of a circumboreally synchronized pattern of seed crop fluctuations in certain high-latitude tree species and a resulting pattern of southward eruptions of birds dependent upon these foods [50, p. 569].

More recent analyses support the existence of synchrony in seed production by boreal trees over sub-continental geographic distances [51,52], but not the intercontinental scale proposed by Bock and Lepthien.

A third important study during this time period was Waller's 1979 theoretical work developing life-history models focusing on the costs of delayed reproduction indicating that masting should be restricted to long-lived populations with high adult survivorship [53]. Last but not least was Silvertown's 1980 classic paper bringing together 59 datasets on seed production and seed predation to test Janzen's hypothesis that the ultimate driver of masting behaviour is primarily predator satiation [54], a hypothesis that continues to enjoy considerable, albeit not exclusive, support today.

## 3. Globalization and the era of the masting specialist (1981–2002)

The majority of the ecological works cited above were done by botanists, ecologists or (in the case of avian eruptions) ornithologists whose study of masting behaviour was a sideline to their broader interests in population ecology and life-history theory. This remains true today, as many of the workers publishing in this field and in this issue (myself included) come from other fields and in some cases still consider their work on masting secondary to other interests. Starting less than a decade after Silvertown [54], however, the field of masting studies had grown to the extent that masting specialists—researchers making this phenomenon a primary focus of their research—emerged. This was also the decade when masting studies greatly expanded globally and taxonomically. Previously, almost all data were from North American or Northern European trees—primarily the UK and Scandinavia—and typically conducted and discussed with a forestry focus. Starting in the late 1980s, studies from Japan [55–58], Korea [59] and New Zealand [45] became available, a globalizing trend that continues today with important studies and masting specialists currently working not only in those countries but also in Argentina, Austria, Australia, China, France, Germany, Greece, Italy, Spain and more, many of whom have contributed to this issue.

Norton and Kelly's 1988 paper [45] deserves special mention, not only because it analysed long-term masting data from a Southern Hemisphere species (the New Zealand rimu *Dacrydium cupressinum*), but because it was the first to clearly articulate alternative hypotheses for mast fruiting and explicitly discuss the hypotheses and predictions of different potential drivers of this phenomenon. These were divided into resource matching—the idea proposed originally early in the twentieth century by Klebs [19] that annual variation in the size of seed crops match annual variation in some limiting resource—and (expanding Janzen's earlier insight [44]), several economies of scale, including predator satiation and wind pollination, by which plants benefit by producing occasional large episodes of reproduction rather than regular small ones. This fundamental distinction between resource matching—a null hypothesis requiring no selective benefit of variable seed production—and hypotheses based on economies of scale—which necessitate an evolutionary driver—remains key to the current understanding of the ultimate factors potentially driving masting [60]. Dave Kelly, one of the first true masting specialists, subsequently authored a key review [43] and, building on earlier work by Alan Mark from the 1960s [61], embarked on an influential and wide-ranging study of mast seeding in *Chionochloa*—a genus of primarily New Zealand tussock grasses—that continues today.

The 1990s and early 2000s saw several important advances that further raised interest in the field of masting studies. Lalonde & Roitberg [62] developed a dynamic optimization model examining the conditions under which seed predators and parasites can select for masting behaviour in a non-masting ancestor. Particularly influential was the work of Isagi *et al*. [63] presenting in detail the resource budget model for mast fruiting, which proposed that large mast years depend on stored reserves exceeding a threshold combined with the need for outcrossed pollen synchronizing seed crops among plants. As such, the resource budget model unites the evolutionary advantages of variable reproduction with the proximate causes of synchronous reproduction. The Isagi *et al*. model provided the foundation for subsequent theoretical work by Aikiko Satake and Yoh Iwasa demonstrating how stored resources in conjunction with pollen limitation can drive synchronized reproduction over large geographic regions [64–66].

On the empirical side, Ostfeld and colleagues in forests of the Northeastern United States provided a dramatic demonstration of the ways that masting is a keystone component of ecological communities by showing that variable acorn crop size drives a chain reaction linking deer populations, ticks and Lyme disease along with mouse populations, ground-nesting birds and gypsy moths (*Lymantria dispar*) [67–70]. Work by my own group provided an early illustration of the large geographic scale over which masting behaviour can occur [51].

This era was also notable for several large-scale analyses conducted in order to better understand the life-history, temporal and spatial correlates of masting behaviour [26,27,42]. The early 2000s also saw the first working group devoted to masting behaviour, organized by Victoria Sork, Dave Kelly, and Andrew Liebhold at the National Center for Ecological Analysis and Synthesis in Santa Barbara, California, USA (https://www.nceas.ucsb.edu/workinggroups/evolutionary-causes-and-ecological-consequences-mast-seeding-plants), a group endeavour that is currently being reprised as a Long Term Ecological Synthesis project, again in Santa Barbara, led by Jalene LaMontagne, Elizabeth Crone and Miranda Redmond (https://lternet.edu/stories/2021-synthesis-awards/).

## 4. Masting studies in the twenty-first century

**Figure 3.**
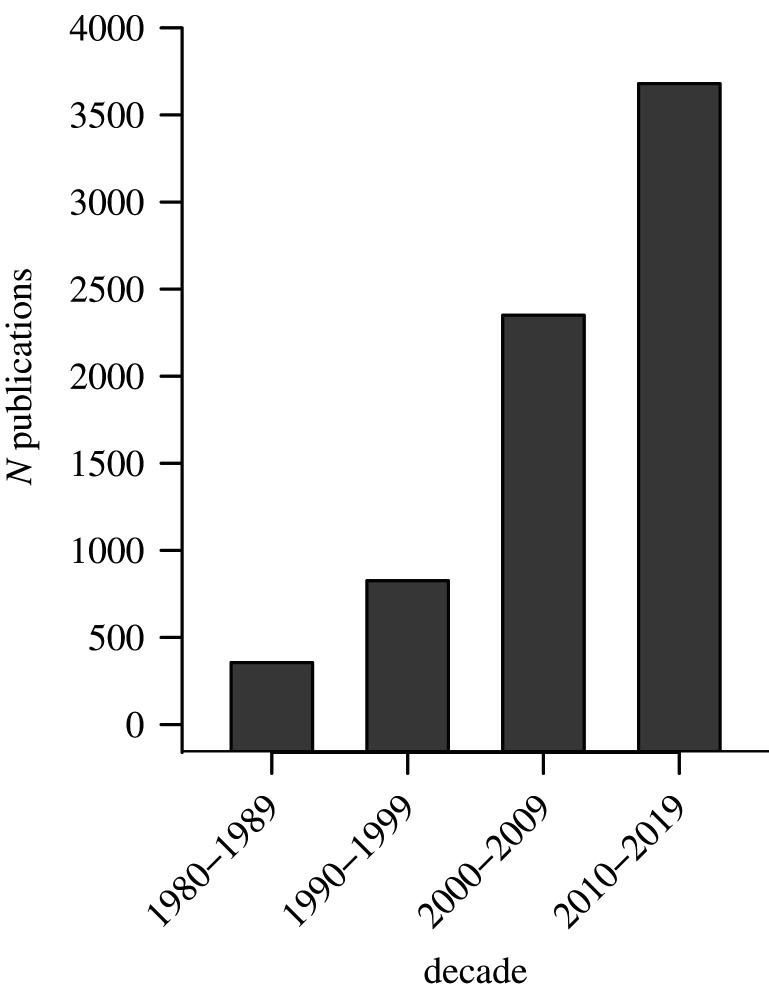
The number of results for ‘mast fruiting’, ‘mast seeding’ or ‘masting behaviour’ in Google Scholar by decade.

The field of masting has exploded since the landmark papers of Janzen and Silvertown. Using the search terms ‘masting behaviour’, ‘mast fruiting’ or ‘mast seeding’, the number of results devoted to masting studies has been growing exponentially over the four decades from 1980–1989 to 2010–2019 (figure 3). Indeed, the number of papers published in 2019 alone (350) nearly matches the number published during the entire decade of 1980–1989 (356). As demonstrated by the breadth of papers in this issue, masting studies have matured to become an active ecological discipline unto itself. Meanwhile, the goal of assembling a comprehensive database of mast fruiting continues, most recently with efforts by Ian Pearse, Jalene LaMontagne and myself, and by the MAST-NET project led by Andrew Hacket-Pain, Andrew Tanentzap and Peter Thomas.

Yet, many basic questions remain unresolved. This includes the semantic issue of whether the term ‘masting’ should be used at all, a conundrum raised by Herrera *et al*. over 20 years ago by analyses indicating that annual variability of seed output among putative masting species exhibited a unimodal distribution that did not depart significantly from normality [42]. (Despite obtaining a similar result in a concurrent analysis [26], I remain a strong advocate of retaining the term ‘masting’, as it offers a clear way to emphasize the focus of the study and thus facilitates it being found and read by those interested in this phenomenon.) An even more basic problem is that of measuring variability itself. Traditionally, the coefficient of variation (CV; standard deviation divided by the mean) has been used to quantify and compare masting behaviour across populations. Unfortunately, CVs suffer from several statistical shortcomings, including a bothersome dependence on the mean and sensitivity to rare events [71]. At least two alternatives have been proposed, the ‘proportional variability index’ (PV) [72] and the ‘consecutive disparity index’ (*D*) [73]. Alas, these alternatives come with their own problems, which are as yet neither as thoroughly explored nor understood as those of CVs. As a result, most authors continue to use CVs as an index of masting, and the problem of how to avoid any bias when comparing variability in annual seed output across populations remains unresolved.

An additional set of fundamental questions focuses on the ambiguities of Janzen's [14] original definition of masting. How synchronized must annual seed output be among individuals within a population, and how large must that population be in order to qualify as masting? Because a majority of seed production data reported in the literature has been taken on a site, rather than individual plant level, variability of seed output among individuals has been relatively understudied. Wide differences among individuals clearly exist, however, and careful analysis of such differences can yield important insights into the relationships among resources, weather, seed production and growth [74]. By contrast, there has been more effort devoted to understanding the extent of the population over which masting takes place, an issue that is usefully quantified by spatial synchrony analysis [75]. Subsequent to Bock & Lepthien's [50] suggestion that such spatial synchrony was intercontinental in geographic scale, studies have confirmed cases of intraspecific spatial synchrony among populations separated by distances upwards of 1000 km [51,52,76], as well as cases in which seed output is interspecifically spatially synchronous [77–79]. The factors driving such dramatic spatial synchrony are not always clear, although spatially correlated environmental conditions (known as the Moran effect; [80,81])—i.e. weather—flowering phenology, pollen coupling and environmental ‘vetos’ limiting resource investment in reproduction are all potential suspects [65,77,82].

More generally, much of the current broad interest in masting remains focused on understanding the proximate mechanisms and ultimate evolutionary factors driving variable seed production. Regarding the former, the resource budget model, although commonly invoked, remains largely untested [83], with the notable exception of Crone *et al*.'s [84] experimental work providing support for the model in the perennial herb *Astragalus scaphoides*. Among other things, the resource budget model provides a potential answer to the critical question of why masting populations do not make smaller seed crops of the same size each year rather than varying annual investment in reproduction, thus focusing attention on the drivers of variance, rather than absolute investment, in reproduction [85].

A particularly pervasive problem is determining what, exactly, the roles of weather are in masting behaviour. Weather directly affects physiological processes that affect the resources available for reproduction and potentially acts as an environmental veto reducing reproduction in particular years [60,86,87]. What remains at issue is whether, at least in some species, weather can also act as an arbitrary cue that enhances fitness by generating synchrony, as recently suggested by Kelly *et al*. [37]. The answer to this question has relevance to how climate change is likely to affect masting behaviour [88–93]. As for the factors driving masting, the relative importance of predator satiation remains of interest [94,95], as are the alternative evolutionary hypotheses of pollination efficiency and environmental prediction [28,60,96] and, at least in some cases, the possibility that variable seed production is *not* a consequence of selection but rather the result of resource matching.

In addition, masting continues to attract the considerable interest of ecologists from other fields. It is currently possible to envision a day when not only the evolutionary factors selecting for masting, but the ways that resources, environmental conditions and pollen coupling interact with molecular and physiological mechanisms to result in variable seed production and synchronization of reproduction in plants have been clearly articulated, at least for a few species [97–99]. Nonetheless, masting will continue to play a pivotal role in ecological studies due to the many ways that the extraordinary resource pulses resulting from mast fruiting affect communities [100]. As a result, this issue is likely to be the first of many that focus on this complex, fascinating and evolutionarily important phenomenon.

## Data accessibility

This article has no additional data.
